# From Field to Waste Valorization: A Preliminary Study Exploring the Impact of the Wine Supply Chain on the Phenolic Profile of Three Sardinian Pomace Extracts

**DOI:** 10.3390/foods13091414

**Published:** 2024-05-04

**Authors:** Ines Castangia, Matteo Aroffu, Federica Fulgheri, Rita Abi Rached, Francesco Corrias, Giorgia Sarais, Gianluigi Bacchetta, Francesca Argiolas, Maria Barbara Pinna, Mariano Murru, Maria Letizia Manca, Maria Manconi, Amparo Nácher

**Affiliations:** 1 Department of Life and Environmental Sciences, University of Cagliari, University Campus, S.P. Monserrato-Sestu Km 0.700, 09042 Cagliari, Italy; matteo.aroffu@unica.it (M.A.); federica.fulgheri92@unica.it (F.F.); r.abirached@studenti.unica.it (R.A.R.); francesco.corrias@unica.it (F.C.); giorgia.sarais@unica.it (G.S.); bacchet@unica.it (G.B.); mlmanca@unica.it (M.L.M.); manconi@unica.it (M.M.); 2 Argiolas SpA|Via Roma, 28/30, 09040 Cagliari, Italy; francesca.argiolas@argiolas.it (F.A.); barbara.pinna@argiolas.it (M.B.P.); m.murru@argiolas.it (M.M.); 3 Department of Pharmacy and Pharmaceutical Technology and Parasitology, University of Valencia, 46100 Valencia, Spain; amparo.nacher@uv.es; 4 Instituto Interuniversitario de Investigación de Reconocimiento Molecular y Desarrollo Tecnológico (IDM), Universitat Politècnica de València, Universitat de València, Av. Vicent Andrés Estellés s/n, 46100 Valencia, Spain

**Keywords:** polyphenols, antioxidant, winemaking, waste, pomace

## Abstract

The winemaking process generates an annual global production of about 10 million tons of waste consisting of stalks, skin, and seeds. The possible reutilization of wine pomace is strictly linked to its chemical composition. In this preliminary study, three different Sardinian white grapes (Malvasia, Vermentino and Nasco) grown in the same area were evaluated through a whole wine production chain. To reduce environmental impact, all the grapes were treated following the integrated production practice (IPP) strategies. The adopted agronomic methods and the main physico-chemical parameters of the fresh fruits and musts were evaluated. A fully qualitative and quantitative characterization of the phenolic fraction of the pomace extracts was performed by HPLC-DAD after a post-winemaking process. Water and ethanol were utilized as green solvents in the extraction process. Additionally, the entire pomace post-winemaking process was carried out within the winery facilities to reduce energy loss and road transportation. The findings demonstrated that large amounts of beneficial polyphenols are present in pomace extracts, and that the type of grape used, agronomic practices, and winemaking method all influence the quantity and quality of the extracts. The polyphenol concentrations in the Vermentino (28,391.5 ± 7.0 mg/kg) and Malvasia pomace (11,316.3 ± 6.5 mg/kg) were found to be the highest and lowest, respectively.

## 1. Introduction

### 1.1. Background

The impending global challenge facing our society is the urgent need to adopt circular patterns of production and consumption to reduce natural resource depletion, global warming, and environmental pollution. In order to this, numerous initiatives are currently underway, and the European Union considers the circular economy transition an irreversible and essential strategy to create a “sustainable, low-carbon, resource-efficient and competitive economy” [1]. In this way, agri-food by-products and wastes may become valuable resources approaching zero waste if knowingly managed using appropriate methodologies based on their composition [2,3]. Among the agri-food residues, those from grape are relevant as about 76,000 square kilometers of emerged lands are dedicated to grapevine cultivation. Approximately 71% of the produced world grape is used for wine, 27% is used as fresh fruit, and 2% is used as dried fruit [4]. In 2022, the main countries of wine production were Italy (49.8 mhl), France (45.6 mhl), Spain (35.7 mhl), the USA (22.4 mhl), Australia (12.7 mhl), Chile (12.4 mhl), and Argentina (11.5 mhl) [5].

The production chain of the wine is composed by three main stages such as wine growing in the field, the winemaking process, and the waste management post-winemaking [6]. The first step regards the selection of the cultivar, the geographical area, and the agronomic conditions, whereas the second stage is related to the type of winemaking process (white or red winemaking). Finally, the third stage involves the disposal of the remaining waste or their storage after drying and comminution before reutilization. At the end of the winemaking process, the main residue is the pomace, consisting of stalks, skin, and seeds, whose annual global production exceeds 10 million tons [7,8]. 

### 1.2. Pomace Reutilization

Grape pomace may be suitably transformed from useless residue to a useful resource since it is rich in nutrients, especially polysaccharides, dietary fibers and proteins, and micronutrients with antioxidant activity such as polyphenols. Although several studies have been conducted in vitro, these molecules are well known for their beneficial effects on human health [9]. Despite this promising content and the significant annual production of pomace, only a limited part is sustainably recycled, and, nowadays, it is mostly used to obtain spirits, composts, or biogas. On the contrary, the recycling of pomace as a source of macro- and micro-nutrients is undervalued despite the important environmental and economic spin-offs offered by this prospective [10]. There is still a long way to reach the sustainable recovery and functional exploitation of grape pomace as well as implement knowledge on the nutrients, especially polyphenols, contained in this by-product, and on the factors that affect their content. The metabolomics of grape pomace are clearly affected by the various steps of the production chain, influencing the quality and quantity of nutrients, particularly polyphenols, contained within. Consequently, these factors determine the final properties of the derived extracts and their potential applications [10,11]. 

### 1.3. Aim of the Work 

In the present study, the influence of a wine production chain on the quantitative and qualitative polyphenolic profile of three monovarietal grape pomaces was evaluated. All analyses were performed on samples (grape, must, and pomace) deriving from fruits treated according to real working conditions during the wine season. The grape pomace derived from three white cultivars (Nasco, Vermentino and Malvasia) of Sardinia was used. To reduce environmental impact, the grapes were treated following the integrated production practices (IPPs), and the extraction step was performed by using green solvents such as water and ethanol. In addition, to minimize road travel and unnecessary energy consumption, the post-winemaking process (drying, shredding, and packaging) was carried out at the winery facilities. The agronomic and ripening parameters of the grape as well as the physical–chemical parameters and total polyphenol content of the fresh fruits and musts were evaluated and compared. To date, no phenolic characterization of pomace extracts of Malvasia and Vermentino is reported in the literature.

## 2. Materials and Methods

### 2.1. Site Description, Climatic Conditions and Agronomic Practices

The wine cultivars grown in the area of Su Pranu (Malvasia) and Picciau (Vermentino and Nasco) of Iselis farm (Serdiana, Sardinia, Italy) were selected for the study. The vineyards have a plant density of about 5950 plants/hectare, and its predominant topography is characterized by elongated ridges with alternating sequences of coarse sandstones, yellowish arenaceous and silty marls. Terraced alluvial gravels, subject to erosion and fragmentation, and sands overlie these structures. Row spacing ranges from 2.2 to 2.5 m with intra-row plant spacing ranging from 0.8 to 1 m. During the study period, the area receives an average annual rainfall of about 450–500 mm (over 60–62 days) with about 150 mm occurring in the period April–September and two rainfall peaks in November and April. The average annual temperatures reached values of 16–17 °C, among which the maximum in July and August accounted for 32.4 °C and the minimum in January accounted for around 5.0–5.5 °C. The grape cultivars used in the study were grown according to integrated production practices, which, depending on the nature of the problem or the desired objective, adopt a variety of agronomic techniques, including the management of runoff and surface erosion, a reduction in organic matter, the management of soil compaction, microbial activity, defoliation techniques such as Simonit & Sirch, conservative and branching pruning, the planned management of green and productive shoots, palisading, shoot guidance, speminization, manual cluster microenvironment management, and tip topping (Table 1) [12]. The control of grasses was applied to better manage the water resources and promote a greater biodiversity in the vineyard; finally, beneficial, and antagonistic insects were used to control of the main phytophages. A significant reduction in the use of synthetic products for crop protection was addressed by sexual confusion and trapping to monitor the presence of harmful insects and intervene when dangerous thresholds were exceeded. This comprehensive approach significantly reduced reliance on synthetic pesticides.

### 2.2. Sample Collection and Fresh Grape Analysis 

During September/October 2022, Malvasia, Vermentino, and Nasco grapes were collected manually, at ripening, from the field and visually inspected. The grape weight, the number of grapes per plant and the quintals obtained per hectare were assessed. The pH, organic acid and sugar content of the harvested grapes were at once measured using an SMC3 Multiparametric Station (Maselli) (Table 2 and Figure S1 in the Supplementary Materials). For moisture determination, an aliquot of each grape sample was cut into two parts with a knife, weighed (10 g) in a porcelain crucible and dried in an oven at 100 °C until a constant weight was achieved (~24 h).

### 2.3. Winemaking Process

The white grapes were transferred to a vat, mixed with ascorbic acid (0.2 mg/L), and gently pressed using a vacuum pneumatic press (−400 mbar). The resulting musts were separated from the pomace by a draining process, cooled to below 10 °C, clarified, left 24 h in static settling, and transferred to steel tanks for fermentation (18 °C). To start the fermentation process, activators and yeasts were added, and, when the conversion of sugars in alcohol was completed, the wine was transferred to special tanks with controlled temperature (15 °C). The pomace from the winemaking processes was stored in special containers before being subjected to post-winemaking (drying, shredding, and packaging). In the winemaking processes, activators and yeasts were purified pectolytic enzymes and *Saccharomyces cerevisiae*, respectively.

### 2.4. Physico-Chemical Parameters of the Musts

The pH, organic acid, alcohol content, sugar content, malic and lactic acid content and total polyphenol content of the must were measured using a multiparametric system, WineScan SO_2_ (Foss Analytical, Padova, Italy) based on Fourier transform infrared spectroscopy coupled with a mathematical calibration model. 

### 2.5. Chemicals

Cyanidin, peonidin 3-o-glucoside, malvidin-3-o-glucoside, quercetin, quercetin 3-o-glucoside, coumaric acid, gallic acid, syringic acid, epicatechin, catechin, and kaempferol (all analytical standard) were from Merck (Milan, Italy). Methanol, ethanol, and acetonitrile (all LC/MS grade solvents), ortophospforic acid 85%, Folin–Ciocalteu reagent and sodium carbonate were bought from Carlo Erba (Milan, Italy). Water was purified through a Milli-Q system from Millipore (conductivity: 18.2 MΩ cm at 25 °C; Milford, MA, USA). 

### 2.6. Fresh Fruit, Must and Pomace Extracts

The fresh fruits collected just before the vinification were pressed manually in a mortar, and 2 g of sample was dispersed in a mixture of ethanol and water (5 mL, 70:30 *v*/*v*), shacked in a vortex (Reax Top, Heidolph, Schwabach, Germany) for two minutes, subjected to agitation using a rotary stirrer (FALC F205/G, Treviglio, Italy) for 30 min at 18 rpm, and finally centrifuged at 4000 rpm for 15 min (Centrifuge 5810 R, Eppendorf AG 22331 Hamburg, Germany). The musts (5 g) were extracted with 10 mL of the same mixture of ethanol and water and processed as described above for the fresh crushed fruits. All the stages were performed at room temperature and atmospheric pressure.

At the end of the winemaking process, the pomace, treated at an industrial level, was dried in an oven at 40 °C for 48 h and ground using an industrial homogenizer (K55, Electrolux Professional, Pordenone, Italy). The powder (10 g) was dispersed in a mixture (200 mL) of ethanol and water (70:30 *v*/*v*) under constant stirring for four hours and finally centrifuged (4000 rpm for 15 min) to separate the solid phase from the liquid phase. 

### 2.7. Total Polyphenol Content of Crushed Grape, Musts, and Pomace Extracts by Folin–Ciocalteu Method

The total polyphenol content of the fresh crushed grape, musts, and pomace extracts was evaluated by Folin–Ciocalteu assay. In a 10 mL volumetric flask, 100 microliters of the previously obtained hydroethanolic extracts was added along with 1 mL of 20% (*p*/*v*) sodium carbonate solution and 500 µL of Folin–Ciocalteu reagent. The volume was adjusted to 10 mL with water, and the flask was vortexed for 3 min. The obtained solution was incubated for 80 min at room temperature in the dark and read at 750 nm using a spectrophotometer Varian Cary 50. The results were expressed as gallic acid equivalents (mg GAE/kg FW). 

### 2.8. Identification and Quantification of Polyphenols of Pomace Extracts by HPLC 

The identification and quantification of phenolic compounds in the pomace extracts were performed using an Agilent HPLC 1100 coupled to a diode array detector and a computerized data integration system (ChemStation-Agilent, LTS 01.11) [10]. The separation was performed with a Kinetex C18 column (5 μm, 150 mm × 4.6 mm) (Phenomenex, Casalecchio di Reno, Italy) at room temperature. A binary solvent gradient was used: (A) phosphoric acid 0.22 M and (B) acetonitrile and methanol (1/1, *v*/*v*) with the following program: t1 = 0 96% A and 4% B; t2 = 40 min 50% A and 50% B; t3 = 45 min 40% A and 60% B; t4 = 70 min 0% A and 100% B; t5 = 71 min 96% A and 4% B to the end (total run time 82 min). The injection volume was 20 µL, the flow rate was set at 0.3 mL/minutes, and the absorbance was measured at 313 nm for hydroxycinnamic acid derivatives, 360 nm for flavonoids, 520 nm for anthocyanins, and 280 nm for all other phenolic compounds (hydroxybenzoic acids and flavan-3-ols). The molecules were identified as a function of the retention times in comparison with those of the commercial standards and the UV-Vis spectra (Figures S2–S6). Calibration curves were obtained using the external standard method, correlating the area of the peaks with the concentration. Before analysis, the previous grape pomace extracts were diluted 1:2 with methanol and vortexed for 1 min; then, they were injected into the HPLC without further purification. All data are expressed as mg/kg fresh weight (FW). 

### 2.9. Statistical Analysis of Data

Results are expressed as the mean ± standard deviation. Analysis of variance (ANOVA) was used for multiple comparisons of means, and Tukey’s test and Student’s *t*-test were performed to substantiate differences between groups using GraphPad Prism 9 (GraphPad; Boston, MA, USA). The differences were considered statistically significant for *p* < 0.05.

## 3. Results and Discussion

### 3.1. Grape Production

The phenological cycle of the selected cultivars followed the same trend throughout all stages; the budburst occurred in April, the flowering stage occurred in May, the veraison occurred in July, and the harvest was performed in September (Table 1). Among the different species, the opening of the leaf buds happened simultaneously, between 3 and 10 April; Nasco and Malvasia were the earliest (3 April) and Vermentino was the latest (10 April). The flowering was more variable (16 days) among the different cultivars; the first (6 May) was Malvasia and the last (13 May) was Vermentino (Table 1). Veraison occurred between 7 July (Malvasia) and 13 July (Nasco) (Table 1). The growth behavior of grapes affected the harvest period, which was not a phenological stage, as it was not entirely established as a function of the physiological characteristics of the plants but rather by business decisions and winemaking objectives. Due to the delay in the flowering and veraison stages, Nasco and Malvasia grapes had a similar developing process and were collected on the same days (20 and 25 September). Vermentino was the first cultivar to be harvested (8 September) (Table 1). The growth stage of the plants was strongly influenced by the cultivars, thermal and rainfall trends, and the type of soil in which the plants were grown [13]. Considering the proximity of the different involved areas, the meteorological conditions (thermal and rainfall trends) and altitude (140 m above sea level) were not variable. Despite their proximity, the growth soils where the crops grow were different. Indeed, Loc Picciau had three different types of soils and Loc. Su Pranu had two soils. Vermentino grew in a soil with a medium useful root depth (50–100 cm) and a medium available water capacity. The internal drainage was greatly reduced in depth with signs of oxidation, and it was defined as calcareous. Nasco grew in a soil with a more clay-like texture, rich in iron concretions, deeper than 160 cm, with a high water availability. In particular, the grown soil of Nasco had a dynamic water table below the soil, and its movement generated a higher water availability. The Malvasia was collected from the locality of Su Pranu, where the soil was slightly sloping and faced west toward a basin of colluvial deposits on the plateau. The available root depth is 100–150 cm, and the water availability is moderate. 

The collecting parameters of the different grapes at harvest were measured (Table 2). The samples showed moisture levels ranging from 79.4 ± 0.8% (Nasco) to 80.3 ± 0.8% and 80.7 ± 1.5% (Malvasia and Vermentino) (Table 2). Vermentino had the heaviest bunches (270 ± 3 g) and the highest yield per hectare (100 q/ha); this value was almost double those of Malvasia and Nasco (50–67 q/ha) (Table 2). Considering a homogeneous average density of plants per hectare, the higher yield of Vermentino strictly depended on the association of the weight of each bunch and the total number of bunches/plant (270 g and 7). The number of bunches per plant partially affect the final yield, as low bunches may favor the enlargement of the remaining ones, but it especially affected their content. Nasco had the lower values, 50 q of fruits in each ha, bunches weighing 210 ± 5 g and four bunches per plant (Table 2). 

The grape yield and weight of bunches were affected by agronomic practices, which involve a complete understanding of vine growth cycles, but also by the fertility of the buds influenced by the health of the plant, the availability of nutrients in the soil, the cultural practices used, and weather conditions. For its part, the number of bunches per plant was influenced by the selected agronomic activities, especially pruning, which permits controlling the total number and size of bunches per plant [14,15]. The last are also affected by the planting density, which influences competition among plants for resources such as sunlight and nutrients. However, in this study, the planting density was equal for all the studied crops. The content of sugars of the fruits was similar, ranging from 185 ± 40 g/kg, that of Malvasia, to 209 ± 40 g/kg, that of Vermentino, without statistical difference (Table 2). Malvasia had the highest acid content, 7.5 ± 4.0 g/L, which was higher but not statistically different from those of Vermentino (6.4 ± 3.0 g/L) and Nasco (5.4 ± 3.0 g/L). These data were confirmed by pH measurements, as Malvasia had the lowest, 3.1 ± 0.1. 

### 3.2. Chemical Characteristics of Musts from Different Grapes and Vinification Processes

At the end of the ripening and collection, grapes of Malvasia, Vermentino and Nasco were treated following the white vinification, which involved a short and low impacting crushing followed by the pressing and separation of pomace and must [11]. The concentration of alcohol, sugar content, organic acid, malic acid, lactic acid, total polyphenols, and pH of the resulting musts, obtained after the separation from pomace, were measured (Table 3). The measured parameters were influenced by the vinification process and the sampling period. According to Guaita et al., the musts were sampled immediately after the pressing step and before alcoholic fermentation took place (unfermented) [11]. As expected, the alcohol concentration of musts was low, ranging from 0 ± 0.1 to 0.09 ± 0.01 (%*v*/*v*), while the sugar concentration was high, ranging from 238.5 ± 9.0 to 257.3 ± 16.0 g/L (Table 3). For the same reason, the concentration of malic acid was high, ranging from 1.7 ± 0.7 g/L to 2.0 ± 0.4 g/L, since it was converted to lactic acid by malolactic fermentation, allowing an inversion of the corresponding concentrations of lactic acid, which is lower (from 0.02 ± 0.01 to 0.3 ± 0.1 g/L) (Table 3).

This chemical conversion of malic acid into lactic acid by the malolactic fermentation led to a significant change in the pH and aromas of the wine [16]. Lactic acid plays an important role not only in the quality of the wine. Synergistically with organic acids (especially malic and tartaric acids), alcohol and acidic pH exert antibacterial activity and additionally address health benefits at the intestine level, improving lactose digestion and avoiding mucosa irritation [16,17]. In white musts, the average concentration of organic acids was 4.4 ± 0.1 g/L, and the average pH was 3.6 ± 0.1. These values were not affected by the time occurring between the crushing and the pressing–separation of must and pomace. 

### 3.3. Total Polyphenol Content of Fruit

Ethanol is the most common green solvent. Due to its high purity, low price, biodegradability and easy availability, ethanol is widely used as an extraction solvent in different fields [18]. In addition, the association of ethanol and water showed a high affinity for different types of phenolic compounds [18]. The total polyphenol content in the extract from the crushed grape of Nasco was the highest, 341 ± 11 mg/kg, which was followed by those of Vermentino, 306 ± 27 mg/kg (Table 4). Extract from the grape of Malvasia had the lowest content, 200 ± 29 mg/kg (Table 4) [19]. The polyphenol content of the grape extracts was influenced by the type of cultivar and the productivity of the plant, depending on the climate condition and the agronomic practices. During the onset of grape ripening (veraison stage) in July (Table 1), all grapes were exposed to high average temperatures (32.4 °C) combined with low rainfall. Water availability is a critical parameter for the concentration of phenolic compounds. A negative water balance from flowering to harvest, coupled with soils with water deficits, can lead to a reduction in berry size and consequently to a high concentration of phenolic compounds [20]. Ramos et al. studied the influence of climatic conditions and soil composition on the polyphenolic content of Tempranillo grapes in Spain. The results revealed a higher concentration of phenolic compounds in climatic conditions with high temperatures and a significant water deficit [21]. In this study, the temperature was a constant factor for all the vineyards, and the water availability of the soil played a key role. However, all the grapes showed an uneven behavior. Despite Nasco being grown in a soil with high water availability, this grape had the lowest yield (50 q/ha), bunch weight (210 ± 5 g) and number of bunches per plant (4), while it also had the highest concentration of polyphenols among grapes (Table 2 and Table 4). On the contrary, Malvasia grown in a soil with a moderate water availability showed the lowest concentration of polyphenols. These data confirmed the importance of the variety-dependent intrinsic properties of the plant.

### 3.4. Total Polyphenol Content of Must 

The total polyphenol content of the musts, performed by infrared spectroscopy in the factory and confirmed by UV-Vis spectroscopy, was mainly influenced by the starting grape. The total polyphenol content of the musts accounted for 685 ± 86 mg/kg (Nasco), 1088 ± 321 mg/kg (Malvasia), and 961 ± 125 mg/kg (Vermentino), respectively (Table 4). The total polyphenol content of the musts of Malvasia and Nasco followed the same trend as that of the corresponding fresh fruits, whereas the resulting must of Vermentino had a higher polyphenol content than Nasco, whose fruits were more concentrated. The grapes were subjected to a soft pressure of −400 mbar, which was followed by the immediate removal of the pomace from the must. During the vinification of Nasco, the seeds and skins suffered only partial breakage during the soft pressing phase; therefore, the rich content of Nasco partially moves from the grape to the must [22]. The data obtained are slightly higher than those of Onache et al. on the varieties Fetească Regală, Riesling Italian, Sauvignon Blanc, and Muscat Ottonel with values ranging from 563.7 mg/L (Fetească Regală) to 414.0 mg/L (Riesling Italian) [23].

### 3.5. Total Polyphenol Content of Pomace

The extracts of pomace obtained from Vermentino showed the highest concentration of total polyphenol (11,507 ± 284 mg/kg), followed by Nasco (9703 ± 435 mg/kg), and Malvasia (9358 ± 278 mg/kg) (Table 4). The total polyphenol content of grape pomace removed immediately after pressing was influenced by the concentration of the initial fruit and the soft pressing stage. The TPC followed the scheme Vermentino > Nasco ≥ Malvasia (Table 4). The behavior of the extracts from Nasco and Vermentino pomace showed an inverse trend to their musts; the polyphenols that did not migrate to the Nasco must enriched the corresponding pomace and vice versa (Table 4). During winemaking, white grape pomace undergoes a less pronounced process than red grape pomace, resulting in a lower transfer of polyphenols to the must than red grape pomace. Additionally, due to the low permeability of the cell walls and cytoplasmic membranes, as well as the short contact time between the must and the pomace, the polyphenols of the fruit mainly remain in the pomace due to their poor extraction [24]. Data obtained confirmed this finding. The total polyphenol content in pomace was several times more concentrated than the corresponding musts and pulp (Table 4), confirming that pomace (seeds and peel) was the part of the grape most concentrated in polyphenols if compared with juice and pulp [25].

### 3.6. HPLC Characterization of Pomace Extracts

Extracts from pomace of Malvasia, Nasco, and Vermentino had a similar phenolic profile sharing 10 main molecules with the same retention times and UV-Vis spectra (Table 5). All the extracts mainly contained five flavonoids, one hydroxycinnamic acid, two hydroxybenzoic acids, and two flavan-3-ols. The polyphenol content followed the series Vermentino (11,316.3 ± 6.5 mg/kg) > Nasco (9821.1 ± 5.5 mg/kg) > Malvasia (9000.2 ± 4.3 mg/kg) (Table 5) [26,27]. The content of flavonoids, hydroxycinnamic acids and hydroxybenzoic acid was almost the same in all pomace extracts (Table 5). Regarding flavonoids, quercetin 3-o-glucoside was always the compound most concentrated followed by a quercetin derivative and quercetin aglycone. Glucose is the main sugar in fruit skins, and as a result, many phenolic compounds are bound to it. They are usually in conjugated forms with sugar residues through β-glycosidic linkages (O-glycosylated) [28]. Normally, the presence of quercetin as aglycone in pomace is due to the enzymatic hydrolysis of the 3-O-glycosides forms during the winemaking process [11]. The low concentration of quercetin aglycone found in the white pomace can be explained by the absence of the fermentation step of the pomace during the white winemaking. Syringic acid and kaempferol were found in trace amounts [29]. Catechin and epicatechin belong to the class of flavan-3-ols. As reported by Garcia-Lomillo et al. (2017), flavanols are the main polyphenols in white pomace due to the lack of anthocyanins. About 65% of the total flavanols accumulate in the seeds, while in the skin, it increases to 21% [30]. Vermentino was the pomace with the highest concentration of flavan-3-ols (10,834 mg/kg), which was followed by Nasco (9305.4 mg/kg) and Malvasia (8553.7 mg/kg) (Table 5). This study is the first to assess the polyphenolic profiles of the pomace of Vermentino and white Malvasia. The pomace of Vermentino was analyzed only in a blend with other pomace (60% Albarola, 30% Vermentino, and 10% Bosco), whereas Negro et al. reported the phenolic profile of red pomace from Malvasia di Lecce [31]. The qualitative profile was similar to those obtained in this study for white Malvasia, except for anthocyanins, but the quantitative value tended to be lower [32]. In addition, Parekh et al. characterized qualitatively and quantitatively by LC-QTOF-MS an extract of pomace from Nasco determining the presence of gallic acid, catechin, epicatechin, procyanidin B2, and quercetin [33]. However, according to Antonic et al. (2020), the polyphenolic profile of grape pomace reported in the literature by different authors revealed a great variability with values ranging from 2800 to 87,000 mg/kg DW [34]. 

## 4. Conclusions

Pomace extracts contain high concentrations of valuable polyphenols, and their content is strictly related to the different steps of the wine production chain (grape variety selection, agronomic strategies, climatic conditions, and winemaking process). The winemaking process slightly affected the phenolic concentration in the pomace, since during the white winemaking, the waste is subject to soft pressing followed by immediate separation from the must. However, depending on their solubility, some compounds can be transferred from the pomace to the must during the crushing phase, leading to a decrease in the total polyphenol content of the remained waste. In addition, the absence of fermentation mediated by activators and yeasts can lead to an overexpression of the glycosylated forms of polyphenols and a lower concentration of the aglycone forms. The intrinsic properties of the variety and the climatic conditions seem to play a pivotal role in the final concentration of polyphenols. Overall, the results indicate that the extract obtained from Vermentino is richer in polyphenols than those obtained from Malvasia and Nasco pomace. These preliminary results have significant implications, indicating that specific pomaces may have ad hoc advantages as a function of their composition and can be used to manufacture different health-promoting products.

## Figures and Tables

**Table 1:** Geographical locations in Serdiana (Italy) and data of grape growth of the Malvasia, Vermentino and Nasco cultivars.

Cultivar	Location	Flowering	Veraison	Harvest
Malvasia	Su Pranu	6 May	7 July	20 September
Vermentino	Picciau	13 May	12 July	8 September
Nasco	Picciau	10 May	13 July	25 September

**Table 2:** Average weight of the bunches (WB, g), number of bunches per plant (B/P), yield (quintal per hectare, q/ha; q = 100 kg), moisture (%), sugar content (g/kg), organic acid and pH from the different grapevine cultivars at harvest time in the area of Su Pranu (Malvasia) and Picciau (Vermentino and Nasco) of Iselis farm (Serdiana, Sardinia, Italy). Mean values ± standard deviations are reported (n = 6). For each group (weight of the bunches, number of bunches per plant, yield, moisture, sugar, organic acid and pH), different letter indicates values that are statistically different.

Cultivar	WB (g)	B/P	Yield (q/ha)	Moisture (%)	Sugar (g/kg)	Organic Acid (g/L)	pH
Malvasia	^b^ 260 ± 3	^a^ 6 ± 1	^b^ 60 ± 4	^a^ 80.3 ± 0.8	^a^ 185 ± 40.0	^a^ 7.5 ± 4.0	^b^ 3.1 ± 0.1
Vermentino	^a^ 270 ± 3	^a^ 7 ± 2	^a^ 100 ± 6	^a^ 80.7 ± 1.5	^a^ 209 ± 30.0	^a^ 6.4 ± 3.0	^a^ 3.4 ± 0.1
Nasco	^c^ 210 ± 5	^a^ 4 ± 1	^c^ 50 ± 2	^a^ 79.4 ± 0.8	^a^ 200 ± 30.0	^a^ 5.4 ± 3.0	^a^ 3.5 ± 0.1

**Table 3:** Concentration of alcohol, sugars, organic acids, malic acid, lactic acid, and pH of musts from grapevine cultivars (Malvasia, Vermentino, Nasco) grown in the area of Su Pranu (Malvasia) and Picciau (Vermentino and Nasco) of Iselis farm (Serdiana, Sardinia, Italy). Mean values ± standard deviations are reported (n = 6). For each group (alcohol, sugars, organic acids, pH, malic and lactic), different letters indicate values that are statistically different.

Cultivar	Alcohol (%*v*/*v*)	Sugars (g/L)	Organic Acids (g/L)	pH	Malic (g/L)	Lactic (g/L)
Malvasia	^a^ 0.09 ± 0.01	^a^ 249.2 ± 9.1	^a^ 4.4 ± 0.8	^a^ 3.5 ± 0.1	^a^ 1.7 ± 0.7	^b^ 0.02 ± 0.01
Vermentino	^b^ 0.04 ± 0.01	^a^ 238.5 ± 9.0	^a^ 4.4 ± 0.7	^a^ 3.6 ± 0.1	^a^ 1.8 ± 0.5	^a^ 0.3 ± 0.1
Nasco	^c^ 0 ± 0.1	^a^ 257.3 ± 16.1	^a^ 4.5 ± 0.6	^a^ 3.7 ± 0.1	^a^ 2.0 ± 0.4	^b^ 0.04 ± 0.1

**Table 4:** Total polyphenol content (mg/kg FW) of extracts obtained from fresh crushed grape, must and pomace of Malvasia, Vermentino, Nasco grown in the area of Su Pranu (Malvasia) and Picciau (Vermentino and Nasco) of Iselis farm (Serdiana, Sardinia, Italy). Mean values ± standard deviations are reported (n = 3). For each group (grape, must and pomace), different letter indicates values that are statistically different.

Cultivar	Grape (mg/kg)	Must (mg/kg)	Pomace (mg/kg)
Malvasia	^b^ 200 ± 29	^a^ 1088 ± 321	^b^ 9358 ± 278
Vermentino	^a^ 306 ± 27	^a^ 961 ± 125	^a^ 11,507 ± 284
Nasco	^a^ 341 ± 11	^b^ 685 ± 86	^b^ 9703 ± 435

**Table 5:** Polyphenolic profile of Malvasia, Vermentino and Nasco pomace extracts (mg/kg FW). Mean values ± RSD% (n = 3) (^a^ quantified as quercetin). LOQ: limit of quantification. For each polyphenolic profile of Malvasia, Vermentino and Nasco pomace extracts (row), the different letters indicate values that are statistically different.

	Malvasia	Vermentino	Nasco
Flavonoids	mg/kg	mg/kg	mg/kg
Quercetin 3-o-glucoside	^a^ 165.5 ± 6.6	^a^ 154.0 ± 6.1	^a^ 159.9 ± 1.0
Quercetin derivate ^a^	^a^ 14.3 ± 7.4	^a^ 17.6 ± 2.9	^a^ 18.5 ± 3.8
Quercetin aglycon	^a^ 16.6 ± 7.0	^a^ 13.0 ± 1.5	^a^ 12.5 ± 7.1
Kaempferol	^a^ <LOQ	^a^ <LOQ	^a^ <LOQ
Hydroxycinnamic acids	mg/kg	mg/kg	mg/kg
Coumaric acid	^a^ 8.8 ± 6.6	^a^ 7.5 ± 6.4	^a^ 5.9 ± 5.4
Hydroxybenzoic acids	mg/kg	mg/kg	mg/kg
Gallic acid	^b^ 206.8 ± 1.3	^a^ 223.6 ± 7.3	^a^ 234.2 ± 9.2
Syringic acid	^a^ <LOQ	^a^ <LOQ	^a^ <LOQ
Flavan-3-ols	mg/kg	mg/kg	mg/kg
Catechin	^b^ 4732.8 ± 2.2	^a^ 6863.5 ± 3.7	^b^ 5243.4 ± 8.8
Epicatechin	^a^ 3820.9 ± 4.4	^a^ 3970.6 ± 6.2	^a^ 4061.9 ± 4.7
Total polyphenol (FW)	mg/kg	mg/kg	mg/kg
	^b^ 9000.2 ± 4.3	^a^ 11,316.3 ± 6.5	^a^ 9821.1 ± 5.5

## Data Availability

The original contributions presented in the study are included in the article/supplementary material, further inquiries can be directed to the corresponding author.

## Supplementary Materials

The following supporting information can be downloaded at https://www.mdpi.com/article/10.3390/foods13091414/s1. Figure S1. Concentrations of sugars (upper panel) and organic acid (lower panel) of grapevines cultivars (Malvasia, Vermentino, and Nasco) during the ripening. Mean values ± standard deviations are reported (n = 6); Figure S2. Chromatogram of the extract of Malvasia pomace recorded at 360 nm; Figure S3. Chromatogram of the extract of Nasco pomace recorded at 360 nm; Figure S4. Chromatogram of the extract of Vermentino pomace recorded at 360 nm; Figure S5. Chromatogram of the extract of Vermentino pomace recorded at 360 nm vs. analytical standard of quercetin; Figure S6. Chromatogram of the extract of Nasco pomace recorded at 360 nm vs. analytical standard of quercetin 3-o-glucoside.

## References

[1] Cifuentes-Faura J. (2022). European Union Policies and Their Role in Combating Climate Change over the Years. Air Qual. Atmos. Health.

[2] Calisto Friant M., Vermeulen W.J.V., Salomone R. (2021). Analysing European Union Circular Economy Policies: Words versus Actions. Sustain. Prod. Consum..

[3] Kassim F., Thomas C., Bioenergy O.A.-B. (2022). Integrated Conversion Technologies for Sustainable Agri-Food Waste Valorization: A Critical Review. Biomass Bioenergy.

[4] Khan N., Fahad S., Naushad M., Faisal S. (2020). Grape Production Critical Review in the World. SSRN Electron. J..

[5] Organisation Internationale de La Vigne et Du Vin (OIV) (2023). State of the World Vine and Wine Sector in 2022. https://www.oiv.int/sites/default/files/documents/OIV_State_of_the_world_Vine_and_Wine_sector_in_2022_2.pdf.

[6] Baiano A. (2021). An Overview on Sustainability in the Wine Production Chain. Beverages.

[7] Zacharof M.P. (2017). Grape Winery Waste as Feedstock for Bioconversions: Applying the Biorefinery Concept. Waste Biomass Valorization.

[8] Perra M., Bacchetta G., Muntoni A., Gioannis G.D. (2022). An Outlook on Modern and Sustainable Approaches to the Management of Grape Pomace by Integrating Green Processes, Biotechnologies and Advanced. J. Funct. Foods.

[9] Rana A., Samtiya M., Dhewa T., Mishra V., Aluko R.E. (2022). Health Benefits of Polyphenols: A Concise Review. J. Food Biochem..

[10] Beres C., Costa G.N.S., Cabezudo I., da Silva-James N.K., Teles A.S.C., Cruz A.P.G., Mellinger-Silva C., Tonon R.V., Cabral L.M.C., Freitas S.P. (2017). Towards Integral Utilization of Grape Pomace from Winemaking Process: A Review. Waste Manag..

[11] Guaita M., Motta S., Messina S., Casini F., Bosso A. (2023). Polyphenolic Profile and Antioxidant Activity of Green Extracts from Grape Pomace Skins and Seeds of Italian Cultivars. Foods.

[12] Simonit M. (2016). Guide Pratique de la Taille Guyot.

[13] Mercenaro L., Oliveira A.F.d., Cocco M., Nieddu G. (2019). Yield and Grape Quality of Three Red Grapevine Cultivars (*Vitis vinifera* L.) in Relation to Altimetry. BIO Web Conf..

[14] Poni S., Frioni T., Gatti M. (2023). Summer Pruning in Mediterranean Vineyards: Is Climate Change Affecting Its Perception, Modalities, and Effects?. Front. Plant Sci..

[15] Poni S., Gatti M., Palliotti A., Dai Z., Duchêne E. (2018). Grapevine Quality: A Multiple Choice Issue. Sci. Hortic..

[16] Garrido-Delgado R., López-Vidal S., Arce L., Valcárcel M. (2009). Differentiation and Identification of White Wine Varieties by Using Electropherogram Fingerprints Obtained with CE. J. Sep. Sci..

[17] Chidi B., Rossouw D., Buica A.S., Bauer F.F. (2015). Determining the Impact of Industrial Wine Yeast Strains on Organic Acid Production under White and Red Wine-like Fermentation Conditions. South Afr. J. Enol. Vitic..

[18] Nutrizio M., Maltar-Strmečki N., Chemat F., Duić B., Jambrak A.R. (2021). High-Voltage Electrical Discharges in Green Extractions of Bioactives from Oregano Leaves (*Origanum vulgare* L.) Using Water and Ethanol as Green Solvents Assessed by Theoretical and Experimental Procedures. Food Eng. Rev..

[19] Guchu E., Ebeler S.E., Lee J., Mitchell A.E. (2015). Monitoring Selected Monomeric Polyphenol Composition in Pre- and Post-Fermentation Products of *Vitis vinifera* L. Cv. Airén and Cv. Grenache Noir. LWT Food Sci. Technol..

[20] Gutiérrez-Escobar R., Aliaño-González M.J., Cantos-Villar E. (2021). Wine Polyphenol Content and Its Influence on Wine Quality and Properties: A Review. Molecules.

[21] Ramos M., Agronomy F. (2020). Variability in the Potential Effects of Climate Change on Phenology and on Grape Composition of Tempranillo in Three Zones of the Rioja DOCa (Spain). Eur. J. Agron..

[22] Serratosa M.P., Marquez A., Moyano L., Zea L., Merida J. (2014). Chemical and Morphological Characterization of Chardonnay and Gewürztraminer Grapes and Changes during Chamber-Drying under Controlled Conditions. Food Chem..

[23] Onache P.A., Florea A., Geana E.-I., Ciucure C.T., Ionete R.E., Sumedrea D.I., Tița O. (2023). Assessment of Bioactive Phenolic Compounds in Musts and the Corresponding Wines of White and Red Grape Varieties. Appl. Sci..

[24] Kammerer D., Claus A., Carle R., Schieber A. (2004). Polyphenol Screening of Pomace from Red and White Grape Varieties (*Vitis vinifera* L.) by HPLC-DAD-MS/MS. J. Agric. Food Chem..

[25] Pastrana-Bonilla E., Akoh C.C., Sellappan S., Krewer G. (2003). Phenolic Content and Antioxidant Capacity of Muscadine Grapes. J. Agric. Food Chem..

[26] Flamini R., De Rosso M., Bavaresco L. (2015). Study of Grape Polyphenols by Liquid Chromatography-High-Resolution Mass Spectrometry (UHPLC/QTOF) and Suspect Screening Analysis. J. Anal. Methods Chem..

[27] Fernandes A., Raposo F., Evtuguin D.V., Fonseca F., Ferreira-da-Silva F., Mateus N., Coimbra M.A., de Freitas V. (2021). Grape Pectic Polysaccharides Stabilization of Anthocyanins Red Colour: Mechanistic Insights. Carbohydr. Polym..

[28] Visioli F., Panaite S.A., Tomé-Carneiro J. (2020). Wine’s Phenolic Compounds and Health: A Pythagorean View. Molecules.

[29] Dangles O., Fenger J.A. (2018). The Chemical Reactivity of Anthocyanins and Its Consequences in Food Science and Nutrition. Molecules.

[30] García-Lomillo J., González-SanJosé M.L. (2017). Applications of Wine Pomace in the Food Industry: Approaches and Functions. Compr. Rev. Food Sci. Food Saf..

[31] Marino A., Battaglini M., Desii A., Lavarello C., Genchi G., Petretto A., Ciofani G. (2021). Liposomes Loaded with Polyphenol-Rich Grape Pomace Extracts Protect from Neurodegeneration in a Rotenone-Based in Vitro Model of Parkinson’s Disease. Biomater. Sci..

[32] Negro C., Aprile A., Luvisi A., De Bellis L., Miceli A. (2021). Antioxidant Activity and Polyphenols Characterization of Four Monovarietal Grape Pomaces from Salento (Apulia, Italy). Antioxidants.

[33] Parekh P., Serra M., Allaw M., Perra M., Marongiu J., Tolle G., Pinna A., Casu M.A., Manconi M., Caboni P. (2022). Characterization of Nasco Grape Pomace-Loaded Nutriosomes and Their Neuroprotective Effects in the MPTP Mouse Model of Parkinson’s Disease. Front. Pharmacol..

[34] Antonić B., Jančíková S., Dordević D., Tremlová B. (2020). Grape Pomace Valorization: A Systematic Review and Meta-Analysis. Foods.

