# Supplementary material for: From Field to Waste Valorization: A Preliminary Study Exploring the Impact of the Wine Supply Chain on the Phenolic Profile of Three Sardinian Pomace Extracts
Source: Foods. 2024 May 4;13(9):1414. doi: 10.3390/foods13091414 (PMC11083656; doi:10.3390/foods13091414)
Supplement: Supplementary file 1 [file foods-13-01414-s001.zip › foods-2961560-supplementary.pdf]

Supplementary material

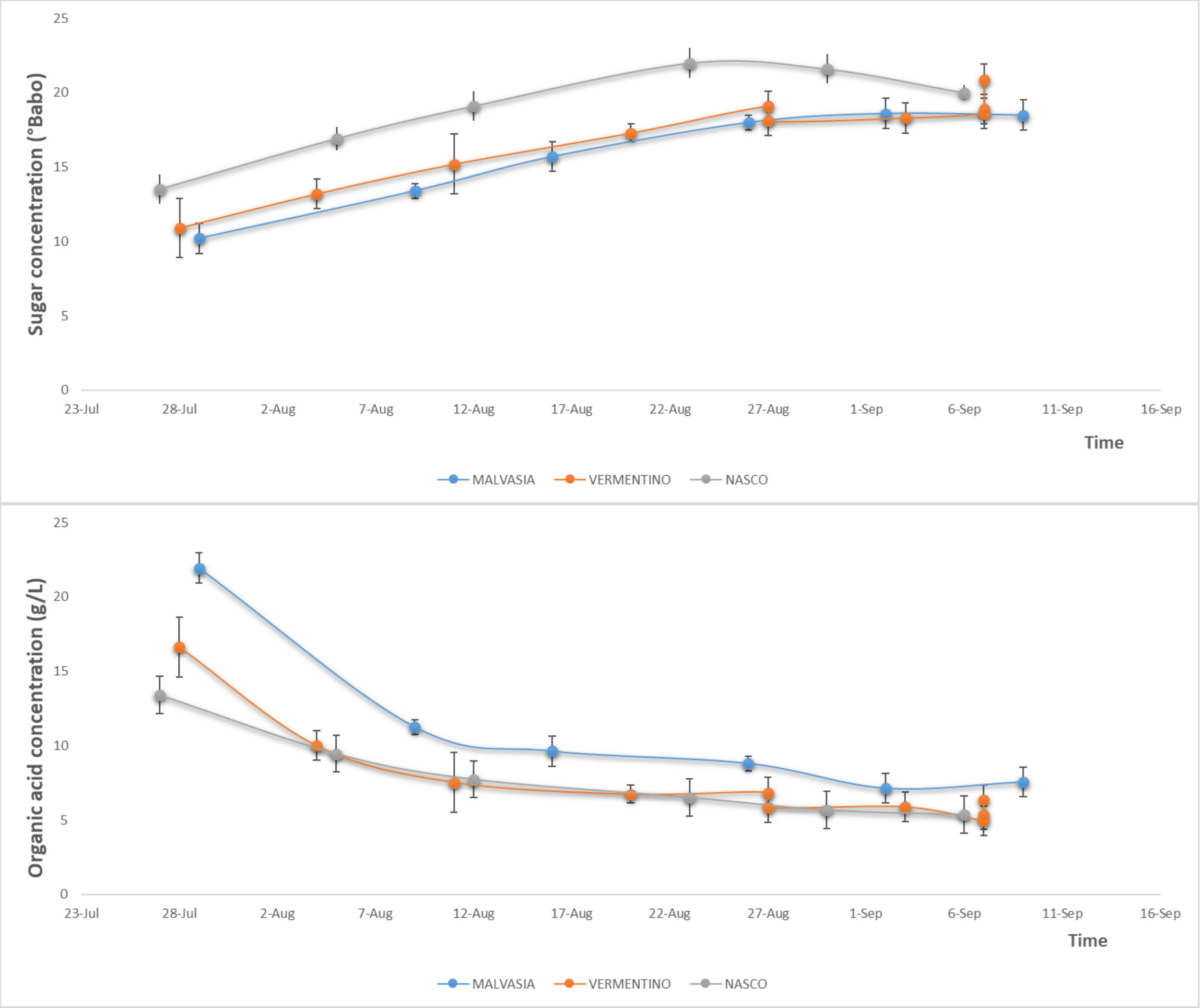

Figure S1. Concentrations of sugars (upper panel) and organic acid (lower panel) of grapevines cultivars (Malvasia, Vermentino, and Nasco) during the ripening. Mean values ± standard deviations are reported (n = 6).

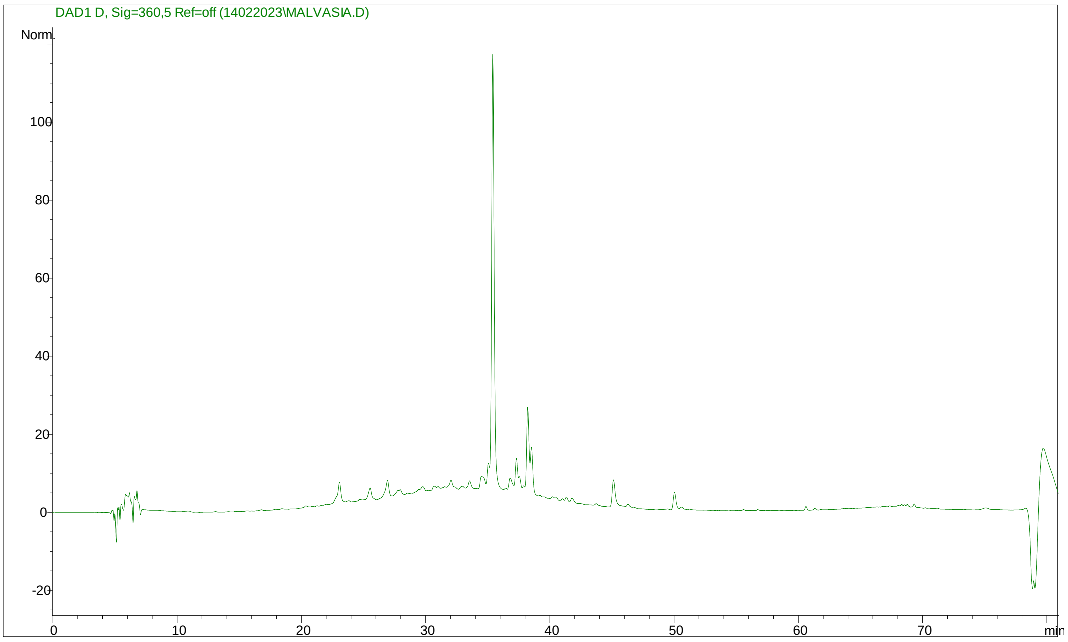

Figure S2. Chromatogram of the extract of Malvasia pomace recorded at 360 nm.

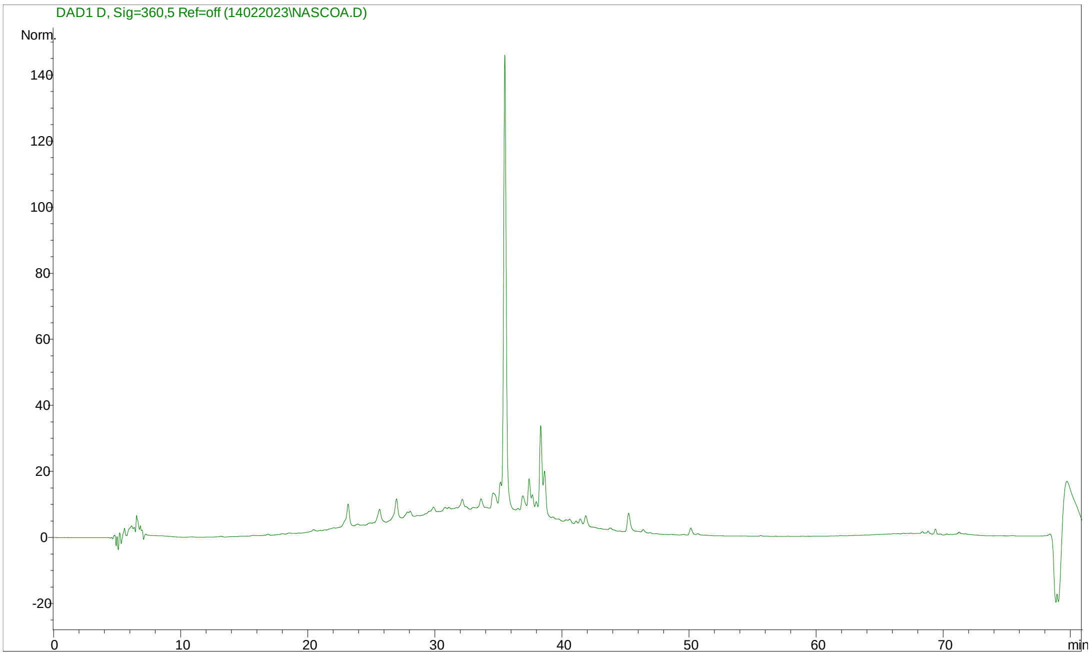

Figure S3. Chromatogram of the extract of Nasco pomace recorded at 360 nm.

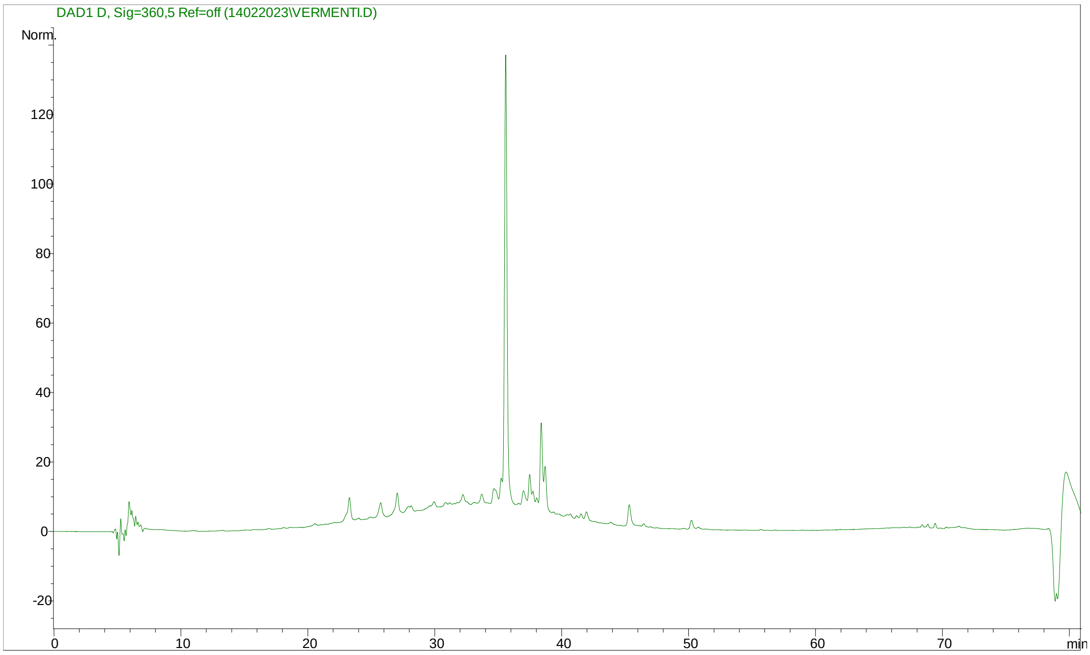

Figure S4. Chromatogram of the extract of Vermentino pomace recorded at 360 nm.

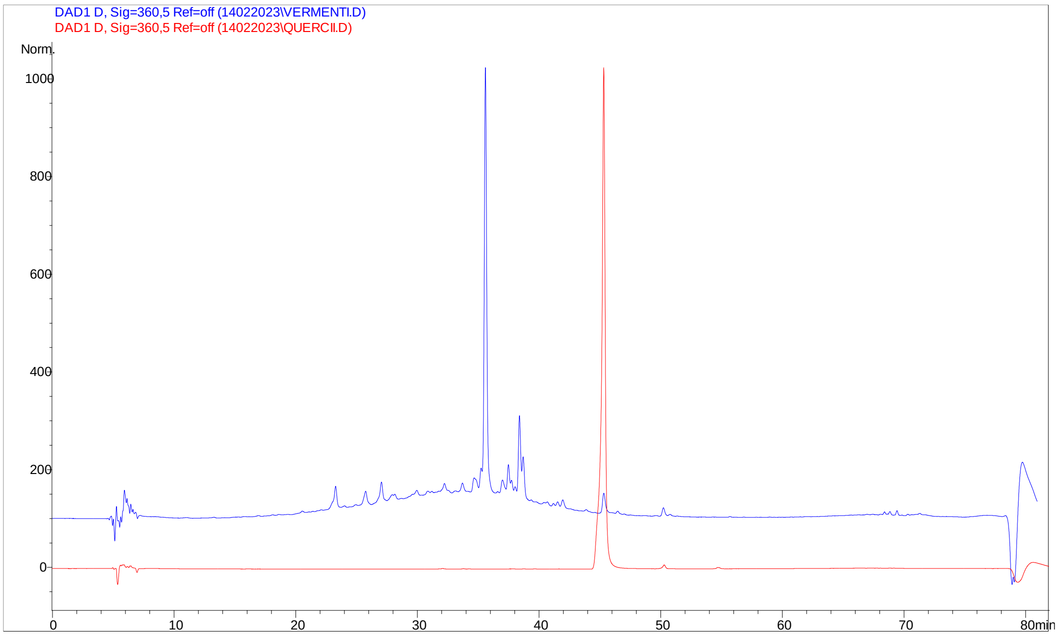

Figure S5. Chromatogram of the extract of Vermentino pomace recorded at 360 nm vs analytical standard of quercetin.

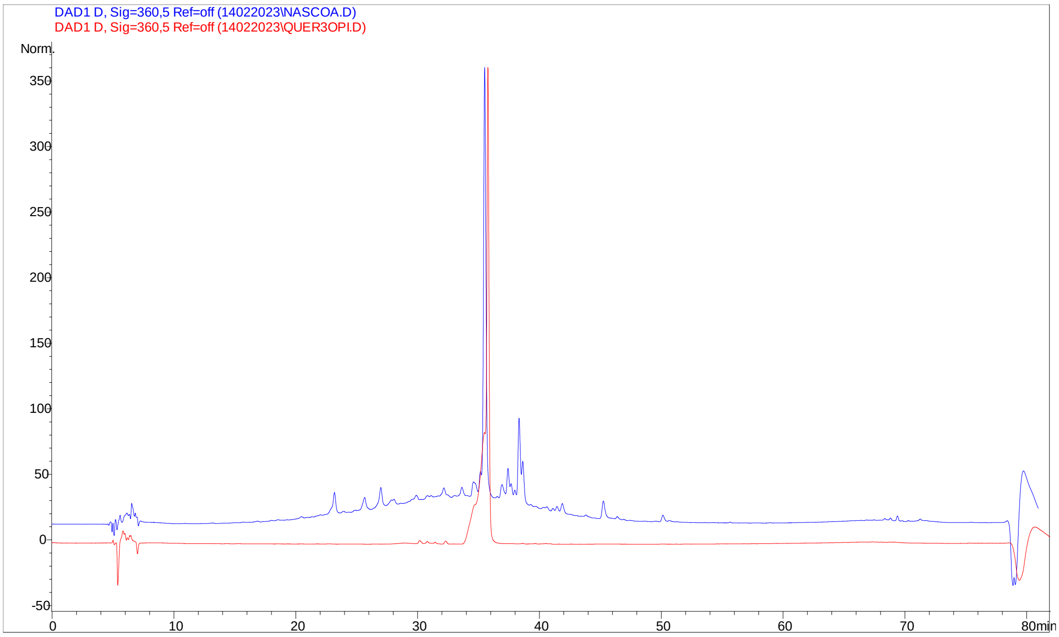

Figure S6. Chromatogram of the extract of Nasco pomace recorded at 360 nm vs analytical standard of quercetin 3-o-glucoside.
